# Prospective Validation of CD-62L (L-Selectin) as Marker of Durable Response to Infliximab Treatment in Patients With Inflammatory Bowel Disease: A 5-Year Clinical Follow-up

**DOI:** 10.14309/ctg.0000000000000298

**Published:** 2021-02-15

**Authors:** Francisco Bravo, Jamie A. Macpherson, Emma Slack, Nicolas Patuto, Julia Cahenzli, Kathy D. McCoy, Andrew J. Macpherson, Pascal Juillerat

**Affiliations:** 1 Maurice E Müller Laboratories, Universitätsklinik für Viszerale Chirurgie und Medizin, Inselspital, University of Bern, Bern, Switzerland;; 2 Gastroenterology, Clinic for Visceral Surgery and Medicine, Bern University Hospital, Bern, Switzerland.

## Abstract

**INTRODUCTION:**

The development of biomarkers to guide management of anti–tumor necrosis factor (TNF) agents in patients with inflammatory bowel disease (IBD) is an unmet need. We developed an *in vitro* blood assay to predict patient long-term outcome with the anti-TNFα agent infliximab (IFX).

**METHODS:**

Patients with IBD were classified according to the shedding of an L-selectin (CD62L) from the surface of their granulocytes in whole blood. CD62L shedding was quantified by flow cytometry before and after drug administration. A clinical data collection from June 2012 to August 2017 with blinded IFX management was aimed at validating the long-term predictive value of this test.

**RESULTS:**

Among 33 patients with IBD (17 Crohn's disease and 5 ulcerative colitis), 22 were predicted functional responders (PFR) and 11 were predicted as nonresponders (NR) according to the *in vitro* test. Five years after study initiation, 72% of PFR were still treated with IFX (vs 27% in the NR group; *P* < 0.05), with a median time spent under IFX of 45 vs 12 months (*P* = 0.019), respectively. Thirty-five medicosurgical events occurred with a median time to first event of 3 vs 30 months (*P* = 0.023), respectively. Our assay was the best independent predictor of staying long term on IFX (*P* = 0.056).

**DISCUSSION:**

An assay-based *in vitro* test for functional blockade of TNFα (CD62L shedding) provides an excellent long-term (at 3–5 years) independent predictor of durable use of IFX in patients with IBD. Testing patients could personalize decision making to significantly reduce costs and risk of adverse events and complications.

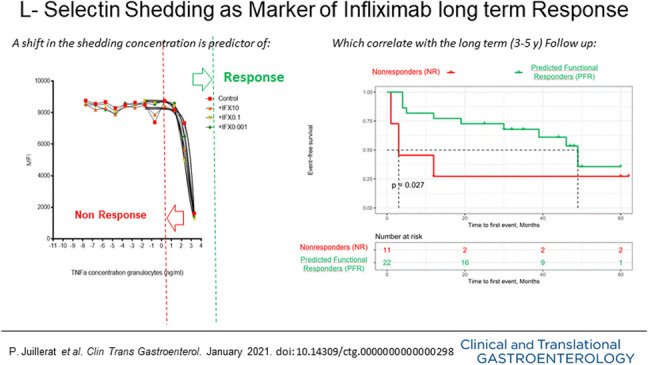

## INTRODUCTION

Inflammatory bowel diseases (IBDs) (mostly Crohn's disease [CD] und ulcerative colitis [UC]) are immunologically mediated chronic inflammatory diseases of the bowel causing significant morbidity for afflicted individuals worldwide (1–5).

The treatment of IBD with biologic agents such as anti–tumor necrosis factor alpha (anti-TNFα) agents has been a dramatic improvement in the management of these patients since the beginning of the 21st century (6–9). In the long term, however, many patients with IBD will progressively lose response (10,11) despite interventions for optimization, such as dose increase and shortened intervals of drug administration (12–14). This loss of responsiveness (LOR) is currently understood in the context of progressively higher serum concentrations of the anti-TNF agent being required to treat high systemic inflammation (15). For example, in severe UC, more anti-TNF immunoglobulins are required (16–18) and significant loss of the therapeutic agent in stool has been reported (19). Increased hypercatabolism or clearance, contributing to LOR, is also favored by to the development of AntiDrug Antibodies (ADA) (13,20,21). This secondary immune response against anti-TNF agents has been widely reported, and the serum levels of ADA have been proposed as a predictor of patient response (22,23). Longitudinal studies have also reported that low drug serum trough levels (TL) and the development of ADA predict clinical LOR in patients receiving anti-TNF inhibitors (15,24–28), leading to the concept of therapeutic drug monitoring (29,30). However, the LOR in some patients could possibly be due to a less anti-TNF driven pathway of inflammation (31), as suggested by the good response rates in the recent ustekinumab trial, a monoclonal antibody directly targeted against interleukin (IL)-12 and IL-23, in TNF-refractory CD patients (32,33). Therefore, a clinical test to measure directly the efficacy of the blockade of the inflammatory effects of TNF on patient primary immune cells is of interest to (i) identify patients likely to develop LOR to anti-TNF agents from the onset of treatment and (ii) tailor the course of treatment based on a biological understanding of each patient's response.

CD62L (L-selectin) is an adhesion molecule on the surface of granulocytes, monocytes, and naive T cells, which is enzymatically cleaved (shedding) on activation of the cells and plays a role in lymphocyte-endothelial cell interactions (34–36). The expression of this molecule on the cellular surface can be quantified by fluorescently labeled specific antibodies to CD62L through flow cytometry (37). For clinical perspective, the “shedding” (release) of this surface molecule is used as surrogate markers for leucocytes activation during chronic or acute inflammation and is stimulated by TNFα. Unlike available commercial tests, which measure static values such as serum infliximab (IFX) TL, this assay is new in its approach because it measures the *in vitro* blood cells' efficient response to IFX administered to the patient. That is equivalent to a functional testing, which investigates changes in innate immunity influenced by the anti-TNF agent, IFX, by modulating L-selectin expression (38). After having published which factors were associated with durable response to IFX in CD (8), our aim was to work in a translational way to validate an *in vitro* test. We hypothesized that our functional test on leucocytes could identify a durable response to anti-TNF inhibitors.

## MATERIALS AND METHODS

### Patients

All patients included in this study (>17 years of age) were in clinical remission (Harvey Bradshaw index [HBI] score or simple clinical colitis activity index [SCCAI] score ≤4) on IFX maintenance therapy in an 8-week interval and had an established diagnosis of CD or UC. Patients were previously identified during visits to the outpatient clinic and after informed consent were included in the SATICC (sensitivity to anti-TNF inhibition in CD and UC) study registry from Bern University, Switzerland. Nonresidents or patients unable or unwilling to provide blood samples or consent were excluded. Exclusion happened also when one of the 2 blood samples (before and after infusion) was missing or not analyzable due to technical problems.

### Clinical data collection

At inclusion, the patient was given an enrollment questionnaire to collect disease activity, extension, extraintestinal manifestations, complications, previous and current treatment (including surgery), as well as laboratory data (C-reactive protein, hemoglobin, and calprotectin). From June 2012 to August 2017, 33 patients with IBD treated with IFX at Bern University Hospital with adequate blood analysis for the response profile were prospectively followed with the same clinical data collected in collection report forms by the treating physicians (A.J.M. and P.J.) during their outpatient clinic appointments at 3 months after enrollment and then every 3–6 months. Clinicians were blinded to the results of the CD62L shedding and therefore took clinical symptoms-based medical decisions (TL and ADA were unavailable). Clinical response was defined using either a significant (≥3 points) reduction of the clinical score (HBI (39) for CD and SCCAI (40) for UC) calculated systematically at different follow-up visits. LOR was defined as a significant increase of the same clinical scores (≥3 points) calculated at different follow-up visits. Finally, the clinical remission was defined as a score of 4 or less in the HBI or SCCAI. Because of the observational prospective design of this study and costs, no endoscopical assessment was required and calprotectin measurement was not systematically performed in 2012.

### Blood collection

Blood samples were collected before and after (within 30 minutes) the anti-TNF antibody infusion at the hospital day clinic at the time of the inclusion of the patient in the study. Two blood samples were taken before the infusion (5-mL citrated blood and serum tube) and 1 blood sample after the infusion (5-mL citrated blood). The citrated blood was used immediately for laboratory analysis (TNFα resistance assay based on CD62L shedding), and patient serum was stored at −80°C for later measurements of drug TL and ADA at the inclusion in the study.

### In-House TNFα resistance assay

**Figure 1.**
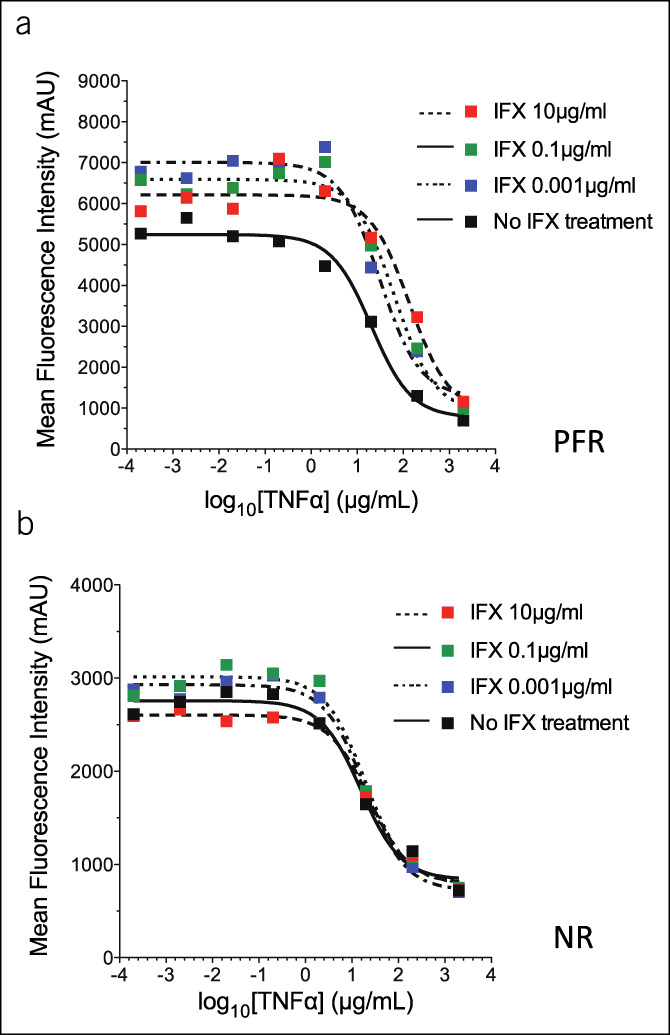
CD 62L granulocyte shedding in 2 different patients as measured by mean fluorescence intensity after incubation of citrate blood with different infliximab (IFX) concentrations. (**a**) Predicted functional responder (PFR). (**b**) nonresponder (NR). TNF, tumor necrosis factor.

Citrate blood was preincubated with 10, 0.1, or 0.001 μg/mL of IFX for 15 minutes at room temperature. The IFX-treated blood was then stimulated with human recombinant TNF (R&D, Minneapolis, MN) with a top concentration of 2 μg/mL or with lipopolysaccharide (LPS) (R&D) with a top concentration of 10 μg/mL and incubated at 37°C, 5% CO_2_ for 45 minutes. After incubation, the stimulated cells were washed with 1% bovine serum albumin/phosphate-buffered saline and stained with 1% bovine serum albumin/phosphate-buffered saline containing 1-μg/mL FITC-anti-human CD62L (Biolegend, San Diego, CA) and 2-μg/mL APC-anti-human CD33 (Biolegend). Cells were then lysed with 10% Fluorescence-activated cell sorting (FACS) lysing solution (BD Biosciences, San Diego, CA) and acquired on a FACSarray bioanalyzer (BD Biosciences). FlowJo FACS analysis software (Tree Star, Ashland, OR) was used to distinguish between monocytes and granulocytes by CD33 expression and side-scatter. The mean fluorescence intensity of the FITC signal was recorded and plotted against the concentration of TNF or LPS used to stimulate the blood sample using Prism GraphPad graphing software (GraphPad Software, La Jolla, CA) (Figure 1). Nonlinear regression curves were fitted and the logEC50 values were calculated, corresponding to the concentration of either TNF or LPS required to induce half the maximal granulocyte or monocyte CD62L shedding (Figure 1). This allowed investigating whether therapeutic TNFα inhibitors prevented the loss of CD62L expression in the blood of patients with CD or UC. A difference of more than 0.5 in the log of the EC50 before and after infusion was considered as a marker of predicted durable response (efficacious TNFα blockade). This test was validated in another study already published by Erdoes et al. (37,41).

### Detection of preinfusion levels of IFX and antibodies directed against the medication

In addition to measuring preinfusion levels of IFX, the measurement of the presence of endogenous antibodies against the prescribed TNF inhibitor was assessed with a drug sensitive assay (Lisa-Tracker Premium IFX kit [Theradiag, Marne la Vallée, France]). All standards and controls were measured in duplicates in a blinded fashion in an external laboratory (Unilabs Laboratories, Cor Lab Ouest Coppet, Switzerland) which has established a validated and standardized procedure used in daily clinical activity in Switzerland. The lower limit of quantification for serum levels of IFX was 0.1 μg/mL. The lower and upper limits of quantification for anti-IFX ATI were 10 and 200 ng/mL.

### Data handling and statistical analyses

Data from the enrollment and clinical follow-up recorded on paper collection report form (questionnaire) and then digitalized using Microsoft Office Professional Plus Excel 2016 (Microsoft Corporation, Redmond, WA). All data were then pooled and processed with Stata MP, v.16.1 (StataCorp LP, College Station, TX) and RStudio (Boston, MA). The Student *t* test, Wilcoxon rank-sum test, and Pearson χ^2^ test were all used in testing data normality between groups. Logistical regression analyses were performed to identify predictive factors of having a good functional blockade of TNFα with IFX (responders) after adjustment for demographics, duration of disease, and baseline comorbidities. To identify factors associated with the need to change IBD treatment strategy, univariate logistic modeling and multivariate logistic modeling were performed. Univariate variables with a *P* value less than 0.20 were used in the multivariate logistic model. Survival analyses for time to first event were calculated according to the Kaplan-Meier method. In our survival analyses, events are defined as an intervention leading to a therapeutic strategy change (IFX interval shortening or dose adjustment, addition of IBD-specific medications, switch within or out of class). Such events were LOR to IFX, clinically defined IBD flare-up, allergic reaction, IBD medication-related adverse event (AE), and autoantibodies against IFX. A *P* value of less than 0.05 was considered to be statistically significant.

### Ethics statement

Licensed physicians at the Inselspital (A.J.M. and P.J.), University Hospital Bern, Switzerland, collected blood samples and clinical data from patients suffering from CD and UC and healthy donors. The study was approved by the Bern Cantonal Ethics Commission (Ref. No. KEK-BE: 132/12), and signed informed consent was obtained from each of the participants.

## RESULTS

### Patient population and clinical characteristics

We recruited 46 patients with IBD in IFX maintenance therapy at our day clinic. They gave consent to participate in the study and provided 104 blood samples (mean 2.26 per patients). However, 8 could not be included due to technical errors, and 5 patients did not reach the required 2 blood samples timepoints collections (before and after infusion) which lead to 33 IBD (25 CD and 8 UC) patients included for the final analysis. The assay showed a functional blockade of IFX (predicted functional response: PFR) for 22 patients (17 CD and 5 UC), whereas 11 (8 CD and 3 UC) had no functional response (nonresponders: NR) to IFX (Figure 1). When comparing both groups, there was slightly more steroid use at inclusion in the NR vs PFR group (*P* = 0.059). However, the duration of previous IFX therapy at admission was not statistically different between *in vitro* PFR vs NR patients (mean 32 ± 27 vs 25 ± 21 months, *P* = 0.57), and none of the other clinical characteristics reached statistical significance (Table 1).

**Table 1.**
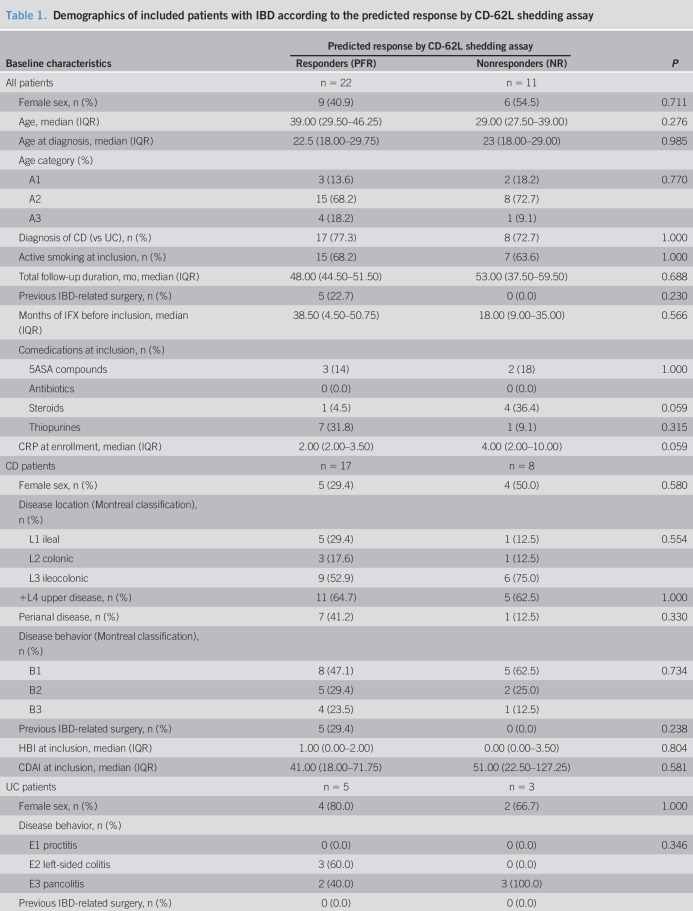

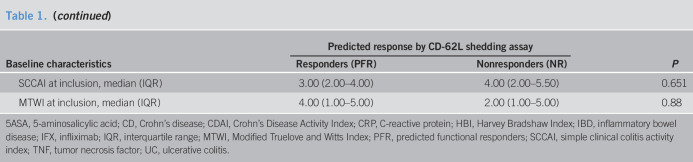
Demographics of included patients with IBD according to the predicted response by CD-62L shedding assay

5ASA, 5-aminosalicylic acid; CD, Crohn's disease; CDAI, Crohn's Disease Activity Index; CRP, C-reactive protein; HBI, Harvey Bradshaw Index; IBD, inflammatory bowel disease; IFX, infliximab; IQR, interquartile range; MTWI, Modified Truelove and Witts Index; PFR, predicted functional responders; SCCAI, simple clinical colitis activity index; TNF, tumor necrosis factor; UC, ulcerative colitis.

### Clinical and biological prospective follow-up

During the 5 years of the study, 7 patients (21%) (3 in the NR and 4 in the PFR groups, respectively) were lost to follow-up. Total mean follow-up time was 46 months (SD ±13.8, range 7–62; 127 patient-years follow-up) and was similar in both PFR and NR groups (resp. 45 and 47 months, *P* = 0.7). Thirty-five medicosurgical events occurred (7 medication-related AEs, 1 treated cytomegalovirus colitis, 21 flares treated with medication, 3 intestinal resections, and 3 operations of fistula) (see Supplementary Figure 1, Supplementary Digital Content 1, http://links.lww.com/CTG/A491), and two-thirds of them during the first 3 years.

Between year 1 and year 3 of follow-up, the proportion of patients who stayed stable without need for intervention (e.g., interval shortening, steroids, or switch of medication) were 16/21 (76%; 1 AE) in PFR vs 1/9 (11%, 2 AEs) in NR (*P* < 0.001). In the PFR group, 4 of the 5 interventions were successful, whereas only half of the 8 interventions of the NR led to clinical improvement or remission.

**Figure 2.**
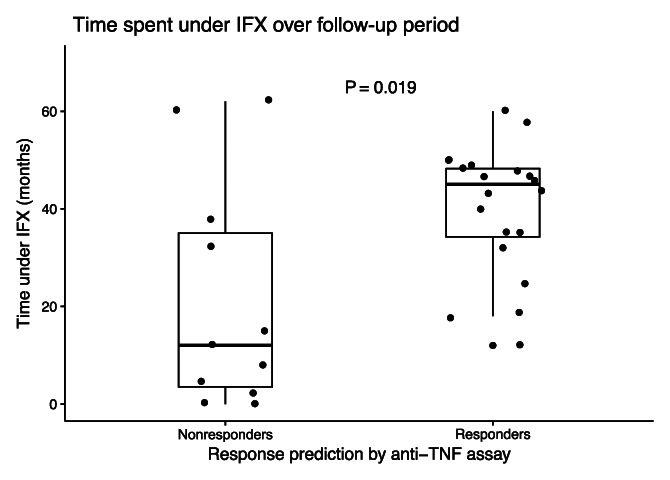
Infliximab treatment duration after inclusion. Dots represent individual patients and the duration of infliximab (IFX) therapy in months, from inclusion to treatment cessation or end of follow-up period. Data are represented as medians ± interquartile ranges (Wilcoxon test). TNF, tumor necrosis factor.

The proportions of patients still under IFX and anti-TNF agents were, respectively, 72% vs 36% (*P* = 0.1) at 3 years and 72% vs 27% at 5 years (*P* < 0.05). The median time spent under IFX therapy after induction was 12 (interquartile range [IQR] 3.5–35) months in the NR groups vs 45 (IQR 34.25–48.5) months in the PFR group (*P* = 0.02) (Figure 2).

The mean delta of calprotectin between first year and second year of follow-up was 1.3 (SD ±85) for PFR and 261 (SD ±255) for NR (*P* < 0.001). The mean calprotectin during the whole first 3 years of follow-up (1 measurement per patient per year available) was 119 (±139 SD) for PFR and 310 (±226 SD) for NR (*P* < 0.001).

### A whole study intervention-free survival analysis

**Figure 3.**
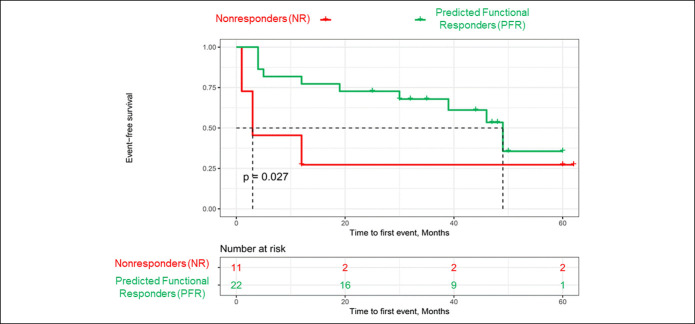
Kaplan-Meier curves of time to first event in months, stratified by response prediction through the anti–tumor necrosis factor assay. *P* value calculated using the log-rank test. NR, nonresponders; PFR, predicted functional responders.

Interventions were defined as any significant clinical situation that led to a change of in the IBD-specific treatment strategy (dose escalation, interval shortening, in-class switch, out-of-class switch, and IFX cessation) during the whole observation period. Median time to first intervention was 3 months (IQR 1.5–12) in the NR group vs 30 months (IQR 9.75–42.75) in the PFR group (*P* = 0.04). Among patients whose disease was inactive at admission, median time to first event was 31 (IQR 19.5–46.3) months in the PFR group and 12 (IQR 12–48) months in the NR group (*P* = 0.6). Intervention-free survival was significantly worse (*P* = 0.027) for patients who were predicted NR by our assay when compared with patients who were predicted responders (Kaplan-Meier; Figure 3). In particular, body mass index, smoking, and concomitant thiopurine therapy or steroid use did not significantly influence the outcome.

### Identification of predictors for interventions and IFX cessation

**Figure 4.**
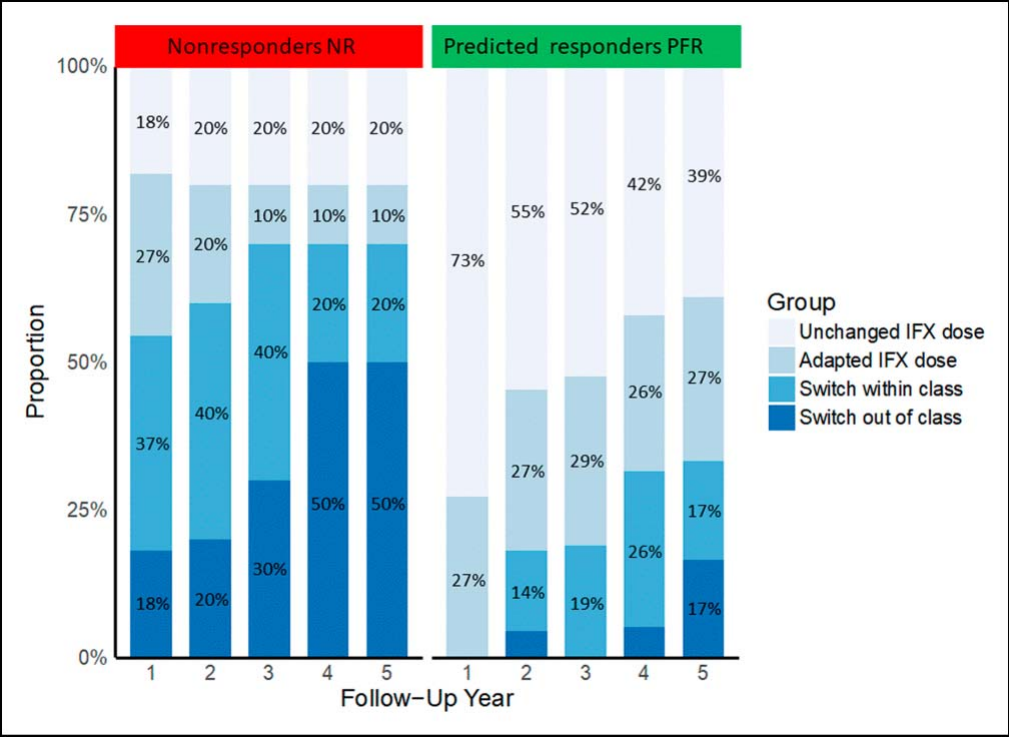
Therapeutic outcomes per patient at the end of each follow-up year, stratified by predicted response groups. IFX, infliximab; NR, predicted nonresponders; PFR, predicted functional responders.

Therapeutic strategies over the 5 years of follow-up are depicted in Figure 4. For our analyses, IBD-related surgery as a therapeutic decision was considered as an out-of-class switch. We performed first univariate and then multivariate conditional logistic regression to identify risk factors for the need to stop IFX at the end of each follow-up year (Table 2). There were no identified significant predictors for the first 2 years of assessment. At 3 years of follow-up, predicted response using our assay was independently and almost significantly associated with the need to discontinue IFX (*P* = 0.056), whereas statistical significance was not reached for the years 4 and 5.

**Table 2.**
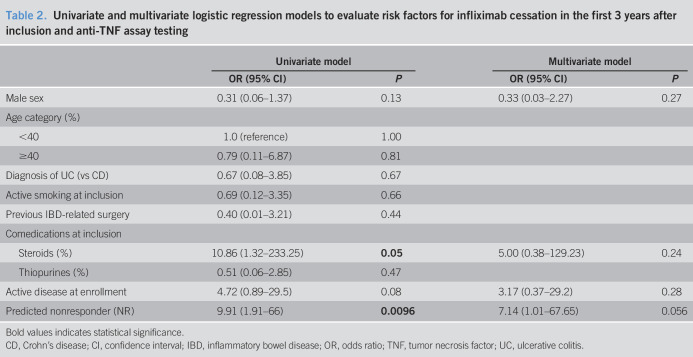
Univariate and multivariate logistic regression models to evaluate risk factors for infliximab cessation in the first 3 years after inclusion and anti-TNF assay testing

Bold values indicates statistical significance.

CD, Crohn's disease; CI, confidence interval; IBD, inflammatory bowel disease; OR, odds ratio; TNF, tumor necrosis factor; UC, ulcerative colitis.

### TL and antibody measurement

TL and ADA measurement could be performed retrospectively (not commonly accessible in 2012) on blood serum samples taken at inclusion in 27 patients (82%; 19 PFR and 8 NR). Four patients developed ADA (2 and 2 in the PFR and NR groups, respectively). Antibodies were detected after 1 and 3.2 years, after performing the anti-TNF assay for the 2 patients in the NR group and, respectively, 2.5 and 2.8 years in the PFR group. There was no significant difference in mean TL values between PFR and NR (3.6 vs 3.8; *P* = 0.9), and only 35% of all patients had a therapeutic TL (>3 μg/mL) despite an appropriate dosage of 5 mg/kg.

As these analyses were performed retrospectively, the clinicians were blinded to TL and ADA values, which led to following interpretation of blinded interventions: In the NR group, an infusion interval reduction was successful in 2 patients who, however, had already optimal TL, whereas this intervention failed in 2 other patients (only 1 with low TL). In the PFR group, all interventions that could have been attempted—based on TL and ADA—have eventually not been performed (clinicians blinded), but were clinically (favorable outcome) not required.

## DISCUSSION

In the present study, we assess a novel *in vitro* laboratory assay for its relevance in predicting durable clinical response of CD and UC patients to IFX, a therapeutic anti-TNFα antibody. Thirty-three patients with IBD in remission on maintenance treatment with IFX were tested and classified according to the results of the assay. A significantly higher number of events needing an intervention (e.g., interval shortening, steroids, or switch of medication) was observed in the predicted NR (45% vs 12% of events per patient year) compared with the PFRs during a 5-year follow-up. This essay also could predict (*P* = 0.056) a durable response to IFX therapy at a 3-year foresight, with a much longer benefit of the TNFα blockade (45 vs 12 months, *P* = 0.02) in the PFR group.

To the best of our knowledge, no other previous study has analyzed functional blockade of TNFα in the field of IBD by using the CD62L shedding assay as surrogate marker. Indeed, this CD62L shedding assay, despite not being so far identified as specific for IBD, allows for direct quantification of the immunologically relevant function of blood granulocytes and monocytes. This suggests an increased value compared with methods quantifying cytokine levels in serum or cell surface phenotypes.

Our assay developed by Emma Slack was also used by Erdoes et al. (41) who could demonstrated that the surgical technique of coronary bypass influences the postsurgical systemic inflammatory response through quantification of the CD62L shedding. This was the first publication, which validated this technique that uses the cleavage of the membrane-bound CD62L molecules as a surrogate parameter for early cell activation.

Interpreting this finding in our patients may be more complex since it potentially reflects as much the systemic inflammation in our patients (to note, the inflammatory markers (Table 1) where similar in both groups) as of the neutrophil responsiveness, the latter being modulated by the drug (IFX) in response to microbial stimulation (LPS molecule) or an inflammatory signal (TNFα) (42). Therefore, our method provides different information a kind of an *in vitro* measurement of an *in vivo* function of the cells of an individual patient using fresh blood samples. The disease activity characterized by the C-reactive protein value at baseline and the use of steroids was different in the 2 groups and could therefore have played a role, as already know from the literature, in anti-TNF inhibitor response. However, these factors did not influence significantly the outcome in the univariate and multivariate analysis.

Other studies pave the way to a tailored IBD therapy based on a specific laboratory test. Wojtal et al. evaluated the efficacy of the various anti-TNFα antibodies and identified the Fc gamma receptor CD64 as central to the mechanism of LOR to IFX, but not to certolizumab pegol (43). Its expression seems to be interferon-gamma induced. Similar and somewhat more complex examples of tailoring therapy to laboratory test results have been already described in the literature (44). Some molecules have been identified through translational research and linked to anti-TNF response or management, such as oncostatine M (45) and TNFα in endoscopical biopsies (46). Serum IL-9 levels were also recently suggested as a new interesting marker of CD activity and linked to response to anti-TNF (47), and the IL-23 pathway activation (with presence of apoptosis-resistant intestinal TNFR2 + IL23R + T cells) is linked to a LOR to anti-TNF agents (31). The Israeli IBD network group developed the first predictive assay based on the analysis-embedded tissue. They could identify predictors of nonresponse to anti-TNFα agents, such as a high proportion of plasma cells in the biopsies and an upregulation of the triggering receptor expressed on myeloid cells (48). This TREM1 differential expression has been confirmed by the Verstockt et al. (49), from Leuven who identified, however, downregulation associated with mucosal healing.

The expression of other candidates genes or group of genes reported associations of TLR2, TLR4, TLR9, TNFRSF1A, IFNG, IL6, and IL1B with response to IFX in IBD (50,51). HLA-DQA1*05 (52), HLA-DRB1 (53), and FCGR3A (54) alleles has also been recently associated with immunogenicity to IFX (50). Finally, a recent single-cell analysis of inflamed tissues from CD patients demonstrates that when a cellular module called GIMATS (IgG plasma cells, Inflammatory Mononuclear phagocytes, Activated T cells, and Stromal cells) is present, there is a strong correlation with a failure of anti-TNF agents to induce steroid-free remission (55).

The strength of our study is that it can explore a new aspect of the response to anti-TNF agent based on a live, cellular-based assay also using blood as opposed to existing *in vitro* assays using drug and ADA detection. The validation of the predictive value of this test over a long, well-phenotyped, clinical follow-up is another important aspect.

The need for fresh blood and rapid analysis (stable results when performed within 3 hours [data not shown]) presents limitations to the daily clinical use of this test. On the other hand, the result of the assay was found to be stable over time within individuals. In selecting patients for inclusion in this study, we were limited to individuals receiving maintenance therapy with IFX at the day clinic. This could arguably present a selection bias toward good responders to the drug. This also implies a specific indication bias for complicated refractory cases (e.g., higher proportion of upper gastrointestinal and fistulizing and previously operated CD). As this study has been performed at a time when therapeutic drug monitoring was not available, we could demonstrate that retrospectively almost two-thirds of the patients were probably underexposed to IFX and not systematically treated with combination therapy, which was less “en vogue” in Europe than in the United States at that time. All this could have impacted on antibody formation; however, the retrospective testing did not show any difference between the 2 groups. Endoscopy and histology as standard evaluations of treatment response were financially not possible in the protocol of our study. However, an endoscopical examination could be performed as standard of care in some patients if required by the treating physician to take his decision.

Sample size is another issue and might be a limitation to the validity and generalizability of our results. In particular, the small number of individuals forced us to combined CD and UC patients when analyzed in logistic regression or survival analyses. The rationale for that was that both diseases have a similar response rate to anti-TNF agents in randomized clinical trials and comparable complication rates.

IFX remains the first-line anti-TNF agent in numerous countries in the management of patients with IBD. Therefore, many patients eventually lose response to IFX (10,56) and experience significant adverse outcomes when flare-ups or medication-related AEs happen, generating increased costs. Literature reviews assert the current need for a better understanding of the immune system of the host in refractory IBD to guide therapeutic strategies toward new classes of molecules (31,57). Our data show that an *in vitro* test reflecting the neutrophil reactivity to anti-TNF agents could help personalizing IBD therapy by identifying patients who would be long-term responders beyond the first year of anti-TNFα treatment. Indeed, all predicted responders still were under anti-TNF treatment 3–5 years after the assay had been performed.

Further investigation on the role of CD62L (L-selectin) is required to better understand its importance regarding other families of molecules used in the treatment of immune-induced inflammation, such as anti-integrins and S1P inhibitors. In example, beside its role in leucocytes migration, L-selectin (CD62L) has been suggested to play a central role in adaptive immune response by initiating lymphocyte recirculation through the lymph nodes (38), whereas in remitting, multiple sclerosis treated with natalizumab-reduced L-selectin (CD62L) expression in T cells in cryopreserved samples has been proposed as a biomarker of pre-PML (progressive multifocal leukoencephalopathy) state (58–60).

CD62L (L-selectin) shedding is the first validated test of functional blockade of TNFα in patients with IBD for long-term prediction of stable disease state under anti-TNF inhibition; this live assay seems to perform better than TL and ADA measurements. This cheap and simple assay could be used in clinical practice to provide a quick answer to the treating physician. This would help him in the decision-making process toward more personalized medicine by rationally identifying patients who might benefit most from anti-TNF treatment or should be early switched to another class of drugs (anti-integrin/anti-IL12/23) to minimize interventions (treatment optimization) and reduce costs and risk of AEs.

## CONFLICTS OF INTEREST

**Guarantor of the article:** Pascal Juillerat, MD, MSc.

**Specific author contributions:** J.C., K.D.M., A.J.M., and P.J.: the original concept (aims, hypothesis, and experimental design) of the project, the supervision, and technical assistance. J.A.M., E.S.: established the setting up and optimization of the initial assay and running patient samples from June to December 2012. P.J.: recruited patients for this study, prepared the ethical guidelines, and was responsible for the clinical data collection, founding, and writing the draft of the manuscript. F.B.: was responsible for the long-term follow-up clinical data collection and processing and writing the draft of the manuscript. All authors had access to the study data, reviewed, and approved the final manuscript and improved its scientific and clinical content.

**Financial support:** This work was supported by a grant from the Ruth and Arthur Scherbarth Foundation, as well as a research grant from Bern University Hospital.

**Potential competing interests:** None to declare.

**Previous presentations:** Part of this work was presented at Digestive Disease Week (DDW) 2018, 2015, and 2014, United European Gastroenterology Week (UEGW) 2015, and the European Crohn's and Colitis Organization (ECCO) congresses 2018 and 2014.

**Study Highlights**

**WHAT IS KNOWN**

✓ Thirty percent of patients will be primary nonresponders to anti-TNFα, and 30%–40% of the responders will lose response over time.
✓ The mechanisms behind TNFα inhibition and loss of responsiveness (LOR) to anti-TNF agents in patients with inflammatory bowel disease (IBD) are poorly understood.
✓ Long-term prognostic markers of therapeutic efficacy are required for ensuring successful clinical treatment.

**WHAT IS NEW HERE**

✓ An assay using CD62L (L-selectin) shedding on the leucocytes surface after LPS or TNFα stimulation showed a high value in predicting a maintained long-term response in anti-TNF–treated IBD patients.
✓ This functional test seems to perform better than trough level and antidrug antibodies measurements as the LOR can be dissected functionally objectively and distinguished from antibody levels and complex formation with the drug.

**TRANSLATIONAL IMPACT**

✓ This assay can be use in clinical practice to optimize decision making and reduce interventions (optimization and LOR) or complications (fistula and stenosis) and surgeries.
